# Investigating the Trend of Mortality, Life Expectancy and Excessive Death with Emphasis on the Role of the COVID-19 Pandemic Period in the Isfahan Province: A Cross-sectional Study of Join Point Regression Analysis 2011–2021

**DOI:** 10.34172/aim.31306

**Published:** 2025-04-01

**Authors:** Maziyar Mollaei Pardeh, Mohammad Hossein Yarmohammadian, Habibollah Azarbakhsh, Golrokh Atighechian, Afshin Ebrahimi, Andishe Hamedi, Mohamad Reza Maracy

**Affiliations:** ^1^Department of Health in Emergencies, Social Determinants of Health Research Center, Isfahan University of Medical Sciences, Isfahan, Iran; ^2^Department of Public Health, School of Health, Ahvaz Jundishapur University of Medical Sciences, Ahvaz, Iran; ^3^Department of Health in Emergencies, Health Management and Economic Research Center, Isfahan University of Medical Sciences, Isfahan, Iran; ^4^Department of Biostatistics and Epidemiology, School of Health, Ahvaz Jundishapur University of Medical Sciences, Ahvaz, Iran; ^5^Social Determinants of Health Research Center, Isfahan University of Medical Sciences, Isfahan, Iran; ^6^Department of Environmental Health Engineering, School of Health, Isfahan University of Medical Sciences, Isfahan, Iran; ^7^Student Research Committee, Shiraz University of Medical Sciences, Shiraz, Iran; ^8^Department of Epidemiology and Biostatistics, School of Health, Isfahan University of Medical Sciences, Isfahan, Iran

**Keywords:** COVID-19, Excessive death, Joinpoint regression, Iran, Mortality rate

## Abstract

**Background:**

Comparing the trends of mortality rates provides valuable insight for policy discussions and promotes awareness of health issues. This study aimed to investigate the changes in mortality rate and life expectancy from 2011 to 2021 and the effect of COVID-19 period on these indices.

**Methods:**

We investigated the data of all-cause deaths between 2011 and 2021 by age group, sex and year using Excel spreadsheets from the National Organization for Civil Registration (NOCR), via collected the census method. Joinpoint regression was used to calculate the trend of mortality rate during the study period.

**Results:**

During the study period, there were 262,708 deaths, of which 148,919 were men (56.68%). The trend of mortality rate in both sexes has been increasing. Life expectancy in men and women decreased from 76.71 and 80.82 in 2011 to 74.43 and 77.53 in 2021, respectively. From 2018 to 2021, there was a significant increase in standardized mortality rate in men (APC=14.74; 95% CI=5.73; 28.65) and women (APC=14.29; 95% CI=4.67; 28.97). However, from 2011 to 2018, we observed a yearly 2.65% decreasing trend in men which was statistically significant (APC=-2.95, 95% CI=-7.67, -0.84). In women, no significant trend was seen.

**Conclusion:**

With the emergence of the COVID-19 epidemic in 2019, the trend of mortality rate and life expectancy changed completely, with additional deaths and decreasing life expectancy. Therefore, prevention, control and treatment of epidemic diseases should be a serious concern of policy makers.

## Introduction

 One of the key factors that needs to be taken into account in social planning is the size and traits of the population, as well as the way they have changed over time and are likely to change in the future.^1^

 According to a World Health Organization report, the coronavirus was first reported in Wuhan, China, in December 2019, and quickly spread to all parts of the world, creating many economic, health, social and political problems.^2,3^ COVID-19 affects the respiratory, cardiovascular, neurological, and urinary systems. It can also cause long-term complications. Therefore, many post-infection deaths can be attributed to the infection. The COVID-19 pandemic has caused many deaths, leading to a decrease in life expectancy.^4,5^ The reported mortality rate from COVID-19 has ranged from less than one percent to 10%, which varied by underlying diseases, demographic differences, and testing strategy.^6^

 Life expectancy at birth is the average number of years a newborn baby is expected to live if the current mortality rates continue. It is a useful indicator to reflect the overall mortality level of a population and summarizes the mortality pattern that exists across all age groups. Life expectancy is an important indicator reflecting various factors, including health programs, healthy and unhealthy behaviors of the general population, causes of death, literacy rates, and gross domestic product per capita. The World Bank calculates the Human Development Index on the basis of these three markers.^7^ In a study conducted in Brazil, based on the total number of deaths reported between 2019 and 2020, life expectancy in 2020 decreased by 1.31 years compared to 2019.^8^

 Iran is currently in the third epidemiological transition,^9^ where the rate of death from infectious diseases is expected to decrease, while the rate of death from chronic diseases is expected to increase.^10^ A review of health and mortality indicators in Iran during the last half century shows that these indicators, including child and maternal mortality, as well as life expectancy at birth, have shown considerable improvement.^11^

 Mortality reports reflect the leading causes of death and their risk factors. In addition, they are useful for determining the burden of disease in a community.^12^ Comparing the trend of mortality rates based on age, sex, and cause across different regions provides valuable insight for policy discussions and promotes awareness of health issues. The purpose of this study was to investigate the changes in mortality rate and life expectancy from 2011 to 2021 and the effect of COVID-19 pandemic on these indices in the Isfahan province.

## Materials and Methods

###  Study Design and Setting

 This population-based cross-sectional study analyzed all-cause mortality data from 2011 to 2021, categorized by age group, gender, year, and city of residence in Isfahan province, using a census approach. The National Organization for Civil Registration (NOCR), Ministry of Health and Medical Education (MOHME) and the statistical center of Iran (2) are reliable information sources for death statistics in Iran. In our study, the source of all-cause mortality data was Isfahan’s NOCR.

 We calculated and reported the mid-year population, number of deaths, as well as the crude and age-sex standardized mortality rates (per 1000) during the study period. Additionally, trend analysis was performed using the Joinpoint regression program.

###  Data Collection

 The statistical center of Iran provided the 2011 and 2016 census data used to gather demographic information categorized by sex and age groups. Population data was recorded using Excel 2016. Since only the 2011 and 2016 census data were available for estimating populations in other years, the Population Analysis Spreadsheets (PAS) software was employed. Additionally, the total number of deaths categorized by age group, sex, city of residence, and year (from 2011 to 2021) was obtained from the NOCR of the Isfahan Province via the census method.

###  Participants 

 The inclusion criteria involved all deaths from any cause that occurred in each calendar year within the Isfahan province, regardless of the registration date. Exclusion criteria were deaths with an unknown age group, deaths that occurred before 2011 but were registered after that year, and deaths of individuals who resided outside the Isfahan province.

###  Statistical Analysis 

 Descriptive analyses were conducted and the results were presented as numbers and percentages. Afterwards, crude and age-sex standardized mortality rates in the different years of the study as well as life expectancy were calculated.

 We used the direct standardization method to account for the impact of age structure on mortality rates. Specifically, it employs the 2013 standard population from the International Network for the Demographic Evaluation of Populations and Their Health (INDEPTH). This method is important because it allows for a more accurate comparison of mortality rates across different populations by controlling for age-related variations, thereby providing insights that are not skewed by age distribution differences.

 PAS was used for estimating life expectancy. This spreadsheet calculates a life table from deaths and population in term of age and has an option for using an independently calculated or estimated infant mortality rate that we calculated in this study. It also produces a life table based on age-specific central death rate (mx) values smoothed by a moving average of the logarithms. The formula for calculating life expectancy is as follows: ex = TX/lx, where ex is life expectancy at age x, Tx is the number of person-years lived after age x, and lx is the number of survivors at age x.

 Joinpoint regression is a statistical method used to identify trends in mortality rates by partitioning an independent variable into intervals and fitting separate line segments to each interval. The points where the segments meet are referred to as joinpoints. To determine the optimal joinpoints, our analysis employed the Bayesian Information Criterion (BIC) and assessed significant changes in the linear slope of the trend. The analysis began with a minimum threshold of zero, representing a straightforward linear trend. Given the limited number of observed data points (0-11), the study restricted the maximum number of joinpoints for statistical testing to one. BIC was estimated to identify the best-fitting model for the data during the study period^13^ as: $BIC\left(k\right)=\ln\frac{SSE\left(K\right)}{n}+2\left(K+1\right)\frac{\ln\left(n\right)}{n}$

 where SSE is the sum of squared errors of the k-joinpoint regression model, n is the number of observations and 2(k + 1) is the number of parameters in the model.

 Also, annual percentage changes (APC) and average annual percentage changes (AAPC) were applied to compare the decreasing /increasing trend of mortality rates. To estimate the APC and AAPC, the following regression model was used:

 APCi = {exp (Bi)-1} × 100 and for AAPC = {exp ((∑WiBi)/(∑Wi)-1)} × 100

 where Bi is the slope coefficient for the ith segment with i indexing the segments in the desired range of years, and Wi indicates the length of each segment in the range of years.^14^

 The trend analysis was done using the joinpoint regression program version 5.0.2 (Statistical Research and Applications Branch, National Cancer Institute).

## Results

 During the study period, there were 262 708 deaths (all causes), of which 148 919 were men (56.68%) and 113 789 were women (43.32%). The average age-sex standardized mortality rate per 1000 was 3.23 for both sexes, 3.64 for men and 2.79 for women. The average crude mortality rate per 1000 was 4.65 for both sexes, 5.20 for men and 4.09 for women. The highest number of deaths and the age-sex standardized mortality rate, with a huge difference compared to other years, were related to 2021 with 34 326 deaths (4.11 per 1000) and 2020 with 32,127 deaths (3.65 per 1000) (Table 1). In terms of age, it is worth noting that 72.67% of all deaths were among individuals who were 60 years old and above. This percentage was higher for women (78.22%) than men (68.42%) (Table 2).

**Table 1:** Population and Number of Deaths in Term of Gender, Year, Crude and Age-Sex-Standardized Mortality Rate (Per 1000) in the Isfahan Province 2011-2021

**Year**	**Population**			**Number of Death **			**Mortality Rate**					
							**Crude**			**Adjusted**		
	**Men **	**Women **	**Total **	**Men **	**Women **	**Total **	**Men **	**Women **	**Total **	**Men **	**Women **	**Total **
2011	2472023	2399685	4871708	12219	9217	21436	4.94	3.84	4.40	3.98	2.88	3.45
2012	2496770	2423306	4920076	11981	8785	20766	4.80	3.63	4.22	3.72	2.69	3.23
2013	2521582	2446999	4968581	12258	9143	21401	4.86	3.74	4.31	3.60	2.66	3.15
2014	2546259	2470561	5016820	11758	9054	20812	4.62	3.66	4.15	3.37	2.62	3.02
2015	2571070	2494251	5065321	11865	9017	20882	4.61	3.62	4.12	3.27	2.48	2.90
2016	2595818	2517879	5113697	12284	9431	21715	4.73	3.75	4.25	3.28	2.52	2.92
2017	2620629	2541570	5162199	12360	9675	22035	4.72	3.81	4.27	3.23	2.58	2.92
2018	2645309	2565129	5210438	12711	10070	22781	4.81	3.93	4.37	3.21	2.58	2.91
2019	2670122	2588819	5258941	13957	10470	24427	5.23	4.04	4.64	3.45	2.61	3.05
2020	2694863	2612446	5307309	18264	13863	32127	6.78	5.31	6.05	4.36	3.37	3.89
2021	2719677	2636139	5355816	19262	15064	34326	7.08	5.71	6.41	4.54	3.65	4.11
Total	28554122	27696784	56250906	148919	113789	262708	5.20	4.09	4.65	3.64	2.79	3.23

**Table 2:** 11-Year Death Due to All Causes by Age and Gender in the Isfahan Province from 2011 to 2021

**Age Group**	**Number of Total Death**			**Number (Percent)**		
	**Men **	**Women **	**Total **	**Men**	**Women**	**Total**
Under 1	2801	3172	5973	4538 (3.65%)	5809 (5.11%)	11247 (4.28%)
1-4	1217	1396	2613			
5-9	743	717	1460			
10-14	677	524	1201			
15-19	2120	754	2874	16195 (10.88%)	6239 (5.48%)	22434 (8.54%)
20-24	2760	923	3683			
25-29	3407	1291	4698			
30-34	3865	1567	5432			
35-39	4043	1704	5747			
40-44	4272	2032	6304	25396 (17.05%)	12730 (11.19%)	38126 (14.51%)
45-49	5133	2615	7748			
50-54	6920	3467	10387			
55-59	9071	4616	13687			
60-64	10950	6425	17375	101890 (68.42%)	89011 (78.22%)	190901 (72.67%)
65-69	12278	8454	20732			
70-74	13228	10916	24144			
75-79	17423	14549	31972			
> 80	48011	48667	96678			
Total				148919 (56.62%)	113789 (43.32%)	262708

###  Mortality Trend

**Figure 1:**
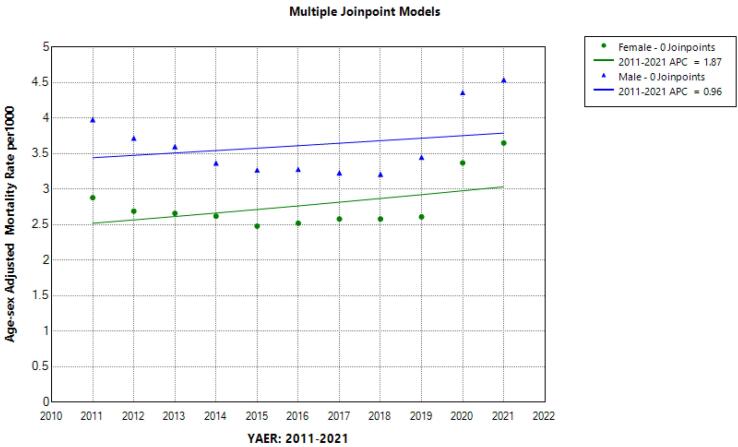
Trend of Age-Sex Standardized Mortality Rate per 1000 in the Isfahan Province 2011-2021. ^*^Indicates that the Annual percent change (APC) is significantly different from 0 at the alpha = 0.05 level

**Figure 2:**
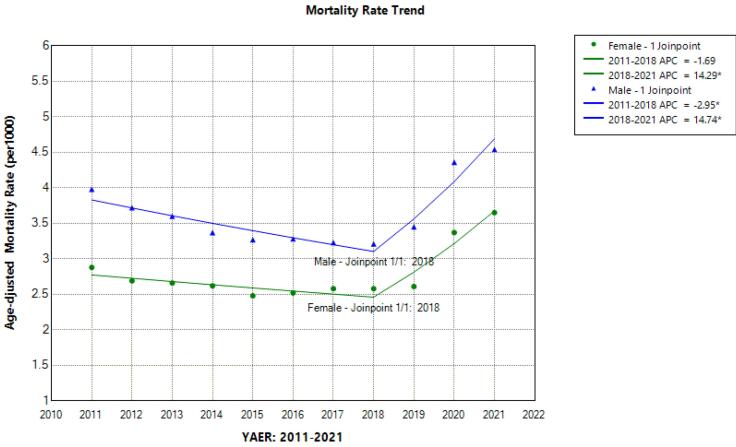
Trend of Age Standardized Mortality in the Isfahan Province between 2011 and 2021 (Trend Modeled with Joinpoint Regression). ^*^Indicates that the Annual percent change (APC) is significantly different from 0 at the alpha = 0.05 level

 The trend of the mortality rate from 2011 to 2021 is shown in Figure 1. The results of the regression analysis of the age-sex standardized mortality rate showed a similar join point in men and women (year 2018) and there were two segments related to 2011-2018 (Segment 1) and 2018-2021 (Segment 2). Accordingly, the APC of the age-standardized mortality rate in both men and women in the first segment has been decreasing from 2011 to 2018; although this decrease was not statistically significant in women, the yearly 2.65% decreasing trend in men was statistically significant (APC male = -2.95, 95% CI = -7.67, -0.84). However, from 2018 to 2021, which is considered as the second segment, there was a sudden, sharp and significant increase in the age-sex standardized mortality rate in both men (APC = 14.74; 95% CI = 5.73; 28.65) and women (APC = 14.29; 95% CI = 4.67; 28.97) (Figure 2). This sharp increase in Segment 2, despite the decrease in the percentage of annual changes in the first segment, caused the AAPC of the age-standardized mortality rate from 2011 to 2021 in men (AAPC male = 2.05; 95% CI = 0.11; 3.34) and in women (AAPC female = 2.85; 95% CI = 0.60; 4.43) to be positive and significant (Table 3).

**Table 3:** Annual Age-Sex Adjusted Mortality Rate, Crude Mortality Rate, and Life Expectancy between 2011 and 2021 (Trend Modeled with Joinpoint Regression)

**Index**	**Sex**	**Segment 1**		**Segment 2**		**2011-2021**
		**Period**	**APC (95% CI)**	**Period**	**APC (95% CI)**	**AAPC (95% CI)**
Adjusted mortality rate (all causes)	Male	2011-2018	-2.95 (-7.67, -0.84)*	2018-2021	14.74 (5.73, 28.65)*	2.05 (0.11, 3.34)*
	Female	2011-2018	-1.69 (-8.61, 0.82)	2018-2021	14.29 (4.67, 28.97)*	2.85 (0.60, 4.43)*
Crude mortality rate (all causes)	Male	2011-2018	-0.44 (-3.71,1.33)	2018-2021	16.08 (8.61, 28.09)*	4.25 (2.68, 5.39)*
	Female	2011-2018	0.23 (-2.35, 1.84)	2018-2021	15.45 (9.11, 26.17)*	4.57 (3.23,5.64)*
Life expectancy	Male	2011-2018	0.44 (0.11, 0.93)*	2018-2021	-2.41 (-4.22, -1.36)*	-0.42 (-0.73, -0.17)*
	Female	2011-2018	0.14 (-0.11, 0.52)	2018-2021	-1.96 (-3.31, -1.15)*	-0.5 (-0.73,-0.30)*

APC: annual percentage change, AAPC: average annual percent change. Data are shown with 95% confidence interval. *Indicates that the APC or AAPC is significantly different from 0 at the alpha = 0.05 level.

###  Life Expectancy

**Figure 3:**
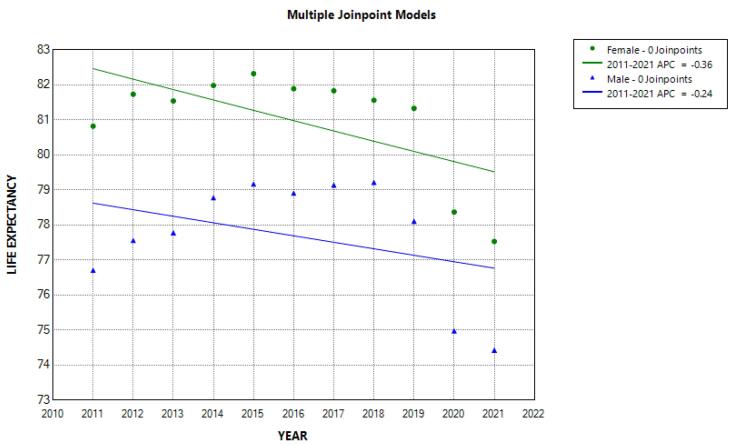
Trend of Life Expectancy at Birth in the Isfahan Province 2011-2021. ^*^Indicates that the Annual percent change (APC) is significantly different from 0 at the alpha = 0.05 level

 The trend of life expectancy of men and women from 2011 to 2021 is shown in Table 4 and Figure 3. Life expectancy in men and women decreased from 76.71 and 80.82 in 2011 to 74.43 and 77.53, respectively in 2021 (not significant). In order to further investigate the trend of life expectancy, using the join point regression analysis, one joint point was found, similar to the analysis of the mortality trend (year 2018), which caused the creation of two segments (2011-2018 and 2018-2021).

**Table 4:** Life Expectancy at Birth by Gender and Year in the Isfahan Province between 2011 and 2021

**Life Expectancy at Birth**			
**Year**	**Men **	**Women **	**Total **
2011	76.71	80.82	78.69
2012	77.56	81.73	79.56
2013	77.78	81.54	79.58
2014	78.78	81.98	80.33
2015	79.17	82.32	80.69
2016	78.91	81.89	80.35
2017	79.14	81.83	80.45
2018	79.22	81.56	80.36
2019	78.11	81.33	79.71
2020	74.98	78.37	76.62
2021	74.43	77.53	75.94

**Figure 4:**
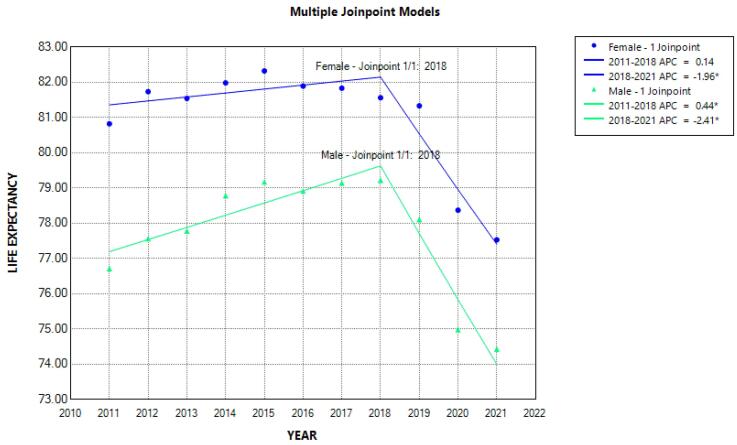
Trend of Life Expectancy in the Isfahan Province between 2011 and 2021 (trend Modeled with Joinpoint Regression). ^*^Indicates that the Annual percent change (APC) is significantly different from zero at the alpha = 0.05 level

 Join point regression analysis showed that from 2011 to 2018, the trend of APC of life expectancy in men was increasing and significant (APC male = 0.44; 95% CI = 0.11; 0.93). However, from 2018 to 2021, it was noticeably and significantly reduced in both men and women. Thus, the percentage of annual changes in life expectancy during these years decreased by 2.41 in men and 1.96 in women (Figure 4). For this reason, the AAPC in life expectancy from 2011 to 2021 was decreased in men (AAPC male = -0.42; 95% CI = -0.73; -0.17) and in women (AAPC female = -0.50; 95% CI = -0.73; -0.30) (Table 3).

## Discussion

 The study showed how the trend of mortality and life expectancy in Isfahan was affected by the COVID-19 pandemic between 2011-2021. The trend of mortality and life expectancy during 2018-2021 has deviated from the normal trend before COVID-19. The age-standardized mortality rates in both men and women declined from 2011 to 2018, but from 2018 to 2021, there was a sudden, sharp and significant increase in standardized age-sex mortality in both sexes. Also, from 2011 to 2018, the percentage of annual change in life expectancy (APC) in men increased significantly, but from 2018 to 2021, life expectancy decreased significantly in both men and women. The percentage of annual changes in life expectancy during these years decreased by 2.41 years in men and 1.96 years in women. A study conducted by Razeghi Nasrabad and Sasanipour found that deaths from COVID-19 reduced life expectancy by 1.4 years in 2020.^2^ A study conducted by Yayla Enfiyeci and Çavlin, titled “Trends in life inequality and life expectancy in Türkiye before and during the Corona period,” showed that during COVID-19, men experienced a greater decline in life expectancy than women in youth and adulthood. In contrast, women experienced a higher mortality rate in old age than men, which is consistent with our study.^15^ Also, in a study conducted by Cao et al in 2023, the results showed that global life expectancy at birth decreased from 72.8 years in 2019 to 71.0 years in 2021, reflecting an annual decline of 1.2% during the period of COVID-19 from 2019 to 2021. This decline occurred despite the overall increasing trend observed from 1990 to 2021.^16^ In a study examining the impacts of the COVID-19 pandemic on life expectancy at birth in Asia, the results showed that the pandemic shortened life expectancy at birth by 1.66 years from 2019 to 2021, slightly less than the global average of 1.74 years. Oman, Lebanon, India, Armenia, Azerbaijan, Indonesia, and the Philippines experienced losses of over 2.5 years in life expectancy at birth. The decline in Asia was primarily observed in the 60–79 age group like this study, followed by the 80 + and 45–59 age groups, while the younger age groups (0–4 and 5–14 years) contributed little to the overall decline. Men suffered more deaths than women during the pandemic. Asian countries experienced fewer losses in the second year of the pandemic (2020–2021) compared to the first year (2019–2020).^17^ A study examining the impact of COVID-19 on life expectancy by Marois et al in 2023 found that a 50% prevalence of COVID-19 infections would reduce life expectancy by 3 to 9 years in North America and Europe, 3 to 8 years in Latin America and the Caribbean, 2 to 7 years in Southeast Asia, and 1 to 4 years in sub-Saharan Africa.^18^ Other studies showed that the coronavirus pandemic reduced life expectancy by 1.13 years in the United States^19^ and by 1.94 years in Brazil.^20^ In Japan, life expectancy increased by 2020 and decreased by 0.15 from 2020 to 2021. The change suggests a possible negative impact of COVID-19 on life expectancy. The study also found that a relatively small increase in the COVID-19 pandemic resulted in a significant impact on mortality rates and that there was a negative correlation between changes in life expectancy and mortality rates.^9^ Changes in mortality and life expectancy trends in the post-COVID-19 era may be influenced not only by health factors but also by economic conditions. One study found that severe economic downturns caused by COVID-19 led to increased mortality and decreased life expectancy. Additionally, the unemployment shock resulting from COVID-19 had long-term effects on mortality rates and life expectancy. Unemployment stemming from the economic crisis can lead to increased mortality due to lack of access to preventive health care and changes in lifestyle.^21^ Considering that the age structure of populations is different and the age structure of Iran’s population is middle-aged, the results of the present study are not comparable with studies in other regions or countries. We also observed significant heterogeneities in mortality rates based on age and sex. According to this study, from 2011 to 2021, 262,708 deaths occurred (due to all causes). The mean age-sex mortality rate was 3.64 in men and 2.79 per 1000 for women. In terms of age, 72.67% of all deaths pertained to people aged 60 years and above. The highest number of deaths and standardized age-sex mortality rates by years were in 2020 and 2021 with a very large difference compared to other years. A study conducted by Ghafari under the prevailing additional deaths related to the COVID-19 epidemic in Iran showed an 8% increase in mortality rates at the beginning of the COVID-19 outbreak and the average of additional deaths across the country increased due to the fact that the epidemic was never fully controlled.^22^ Therefore, additional deaths during 2020 and 2021 can be linked to the COVID-19 epidemic. In a 2018 study conducted in Tehran, the prevalence of mortality in men and women before COVID-19 was reported at 56.7% and 43.3%, respectively. The prevalence of COVID-19 was 61.5% in men and 38.5% in women, respectively. Overall, mortality rates in men were higher than women in both pre- and post-pandemic periods, and the prevalence of mortality in age groups aged 50 and above showed a significant increase, which confirms the findings of the present study.^23^ While the crude mortality rate in Turkey remained stable from 2017 to 2019, a significant increase has been observed since 2020, attributed to the impact of the COVID-19 pandemic. Similar to our study, the result of this study showed that the crude mortality rate was higher among those aged 65 years and above.^24^ The pattern of infection and death rates during the COVID-19 pandemic has varied significantly across countries. Surprisingly, wealthier countries with more healthcare resources have experienced higher death rates than less affluent countries like India and other Southeast Asian nations. This may be attributed to better registration and follow-up care programs in these wealthier countries.^25^ The results of the 2016-2020 analysis using data on all deaths among Latinos in California found that in the first seven months of the pandemic, there were 10,316 more deaths among Californian Latinos compared to trends four years earlier: a relative increase of more than 31%. The results also showed that the effects of the pandemic on mortality were far worse for Latinos born in Mexico and Central American countries than for Latinos born in the United States. The difference was worse among those aged 55 to 74. This finding shows the impact of population age structure and vulnerability to death from COVID-19. Among foreign Latinos, men had more additional deaths than women.^26^ A study conducted in tFrance by Fouillet et al in 2020 found that 14 weeks after the onset of COVID-19, the estimated number of deaths was 60% higher than expected, and the estimated number of deaths was 16.6% above the baseline level for the whole of France. Also, the probability of death was significantly higher in men and in the group aged above 65 years.^27^ In Italy, a review of all-cause mortality data from 2015 to 2020 showed that the COVID-19 pandemic was linked to an increase in all-cause mortality rates. This phenomenon has also been observed in other European countries, such as Spain, Britain, Belgium, the Netherlands, and Sweden.^28,29^ Increasing mortality rates, especially in vulnerable groups such as the elderly, lead to major disruptions in human development in countries. On the other hand, the devastating social and economic effects of COVID-19 on human life further reduce life expectancy. Countries whose age structures are aging rapidly, including Iran, are among the most vulnerable to infectious epidemics such as COVID-19.

 The main strength of this study is the availability of data from several years before the pandemic, which allows us to estimate trends in mortality and life expectancy during an epidemic and compare it to deaths during similar non-pandemic periods. It also provides an effective way to study the impact of individual and social factors on additional mortality. However, this study also has limitations. We examined death due to all causes, and changes in life expectancy based on the leading causes of death were not investigated. Socio-economic inequalities can also have a significant impact on the pattern of mortality and life expectancy which were not addressed in this study. The causes of an increase in mortality rates during 2018-2021, such as failure to comply with health protocols, reopening recreational, sports and educational venues, etc., were not investigated. Lack of access to death data from COVID-19 is the most important limitation of this study.

## Conclusion

 Based on the results from this study, from 2011 until before COVID-19, the trend of mortality was decreasing and life expectancy was increasing, but with the emergence of the COVID-19 epidemic in 2019, this trend changed completely with additional deaths as a result of decreasing life expectancy. Therefore, prevention, control and treatment of epidemic diseases should be a serious concern of policy makers.
